# The Ministry of Health and National Baby Week

**Published:** 1919-07-12

**Authors:** 


### MATERNITY AND CHILD WELFARE.

### The Ministry of Health and National Baby Week.

An important conference on this subject was held in the Kingsway Hall on July 1, 2, and 3, and while nothing very new or startling was brought forward, several aspects of this subject were considered and emphasised. Among the speakers were medical officers of health, medical specialists in the various branches of the work, health visitors, both municipal and voluntary, representatives of Boards of Guardians, and many voluntary societies and associations, including the Salvation Army, and all contributed useful views, one effect of which will be the spreading of what the Minister of Health calls "common knowledge" among the general population of these Isles, without which efforts to reduce the infantile mortality will be not only laborious but ineffective.

The proceedings were opened by Dr. Christopher Addison, who gave a forecast of the lines on which the Ministry of Health intends to proceed in regard to the subject of maternity and child welfare, and stated that special attention will be paid to the subject, and that a special department has been created, presided over by Dr. Janet Campbell, for the purpose of considering the problems of maternity and child welfare.

### The Value of Sympathy.

The keynote of the policy of the Ministry of Health might be summed up in the one word "sympathy"; sympathy with the efforts of municipal and voluntary bodies engaged in this important work, and anxiety to help them both by advice and money; sympathy with the mothers; sympathy with the sanctity of the home, and avoidance of undue and petty interference in family affairs, that the coming generation need not fear that they are likely to be "bossed by doctors from the cradle to the grave." He hopes that there will be such a spread of common knowledge among the mothers that they will come voluntarily to the centres to ask for information about their babies, and be treated by the health visitors and others running the centres in a kind and sympathetic way. Dr. Addison also emphasised the importance of gaining the friendly co-operation of doctors and midwives. As regards the constructive work, he advised the multiplying of maternity hostels for normal cases and hospitals for abnormal ones, and homes of rest. The importance of housing and its connection with infantile mortality was also dealt with.

### Infant Mortality.

After the address the remainder of the morning and afternoon sittings were mainly devoted to ante-natal, natal, and neo-natal mortality, and very able papers were read by Dr. Amand Routh and other experts, in which they brought forward cogent reasons to show that contrary to much recent teaching the mortality, which is very high during these periods, can be very substantially reduced by appropriate measures. Among ante-natal causes of death of the child the chief are syphilis and toxæmia of pregnancy—the former completely preventable and the latter largely so. The natal causes of mortality can also be more or less abolished by skilled attention at birth. Taking the neo-natal period as the month succeeding birth, the mortality in this will be largely reduced by a reduction in the mortality of the first two periods—*viz.*, the ante-natal and natal periods. There are, of course, many other causes of mortality in all of them, such as those due to deformities, the causes of which are quite unknown and therefore cannot be removed.

### Industrial Employment of Mothers.

The second day was mainly devoted to the effect of the industrial employment of mothers on infantile mortality, and there was pretty general agreement that neglect of the home while the mother is at work, and the inadequate care she can bestow on it when she comes home fatigued and unfit for domestic duties, has a most deleterious effect on the welfare of the children. As to the remedy there was some diversity of opinion. One view contemplated the necessity of mothers in the future regularly supplementing the family income by going out to work, and the desirability of greatly multiplying crèche-nursing schools and centres where children can be cared for during the absence of the mother. A similar plan was very ably put forward by Miss Lilian Barker, late Welfare Superintendent of Woolwich Arsenal. She, however, found few supporters, and the feeling of the conference was decidedly in favour of keeping the mother at home and having her income supplemented by mother pensions, or by finding some method of assuring the father a sufficient income to provide properly for wife and family. Most people who think seriously of the matter will support this view, and find out some method of keeping the mother at home, which is her proper place.

### The Illegitimate Child.

The third day was mainly devoted to the unmarried mother and the illegitimate child, and one could not help feeling that every one approached this subject much more sympathetically than would have been done twenty-five years ago. It was a most healthy sign of the times to see the Bishop of Kensington on the platform, and to hear his views on this important subject. It is to be hoped that his example will be followed by the clergy generally, and that while not condoning vice they will try to get at the cause of illegitimacy, and, instead of punishing the unfortunate victims, try to remove the cause and prevent its occurrence.

There is only one general criticism, which applies to this as to many other such popular conferences where the lay element pre-dominates, and that is that the members are liable to be carried away by harrowing details and sympathy into what a doctor would call treatment of symptoms instead of causes, and a timely warning was uttered by Dr. Waller on the danger of false deduction on insufficient premises, with the ignoring of the doctrine of evolution. People may say what they like, but nature works for the survival of the fittest. She has plenty of time and is therefore slow in her methods, so slow that the effects are invisible in the lifetime of an individual or even a nation, but her work is none the less sure, and Dr. Waller's advice that, as a corrective to hasty deduction and the application of quack medicines, every person interested in these matters should read Karl Pearson's "Nature and Nurture" was very sound.

